# Optimisation and Assessment of Three Modern Touch Screen Tablet Computers for Clinical Vision Testing

**DOI:** 10.1371/journal.pone.0095074

**Published:** 2014-04-23

**Authors:** Humza J. Tahir, Ian J. Murray, Neil R. A. Parry, Tariq M. Aslam

**Affiliations:** 1 Faculty of Life Sciences, University of Manchester, Manchester, United Kingdom; 2 Centre for Hearing and Vision Research, Institute of Human Development, University of Manchester, Manchester, United Kingdom; 3 Vision Science Centre, Manchester Royal Eye Hospital, Central Manchester University Hospitals NHS Foundation Trust, Manchester Academic Health Science Centre, Manchester, United Kingdom; 4 Faculty of Medical and Human Sciences, University of Manchester, Manchester, United Kingdom; The Ohio State University, Center for Cognitive and Brain Sciences, Center for Cognitive and Behavioral Brain Imaging, United States of America

## Abstract

Technological advances have led to the development of powerful yet portable tablet computers whose touch-screen resolutions now permit the presentation of targets small enough to test the limits of normal visual acuity. Such devices have become ubiquitous in daily life and are moving into the clinical space. However, in order to produce clinically valid tests, it is important to identify the limits imposed by the screen characteristics, such as resolution, brightness uniformity, contrast linearity and the effect of viewing angle. Previously we have conducted such tests on the iPad 3. Here we extend our investigations to 2 other devices and outline a protocol for calibrating such screens, using standardised methods to measure the gamma function, warm up time, screen uniformity and the effects of viewing angle and screen reflections. We demonstrate that all three devices manifest typical gamma functions for voltage and luminance with warm up times of approximately 15 minutes. However, there were differences in homogeneity and reflectance among the displays. We suggest practical means to optimise quality of display for vision testing including screen calibration.

## Introduction

Modern portable tablet computers are ideal for home vision testing. They are relatively easy to program and touch-screen functionality allows great versatility. Spatial resolution is sufficient for testing visual acuity and the dynamic range of luminance allows a reasonable range of contrasts to be presented. Perhaps most important is the acceptance of touch screen technology by older patients who appear able to easily interact with the new devices comfortably. The attraction of the concept is that patients' visual function may be monitored remotely whilst they remain at home, thus reducing the number of hospital visits.

The possibility of assessing visual function at home has been stimulated by the challenges of an aging population and the increasing socio-economic burden of hospital care. A good example is Age Related Macular Degeneration, the leading cause of blindness in the U.K [1]. AMD patients undergoing Vascular endothelial growth factor (VEGF) treatment might avoid some visits to clinic if a reliable method of self testing were available. If sufficiently sensitive and specific, the approach should reduce the burden of unnecessary visits for patients. This would have a major impact on patient independence and quality of life [2].

Two recent studies [3], [4] have evaluated the use of an iPad for testing the contrast sensitivity function and had promising results, demonstrating good reliability and repeatability for the test in laboratory conditions. At present however, we see two issues restricting the large-scale application of home testing. The first is the fact that all vision tests require the accurate control of luminance, size and contrast of targets. Indeed, one of the issues that arose in [4] was the effect of glare on the screen. The second is the validation of the approach; to what extent can patients be relied upon to carry out tests reliably when they are away from a clinical environment? In the present paper we address the first of these concerns by rigorously testing the physical characteristics of the display of three commercially available devices, one of which we have tested in a previous report [5]. The objective of the investigations is to compare two other devices with the iPad 3 and also to describe a protocol that might be used by others to establish the suitability of new tablet computers that will inevitably be introduced in the future.

## Methods

Three devices were tested for this study, an iPad 3 (Apple Inc.) a Google Nexus 10 (Google Inc.) and Galaxy Tab 2 10.1 (Samsung Electronics). Table 1 lists the resolution of the screens, physical sizes and the minimum viewing distance to permit testing 1.0 min of visual angle subtended per pixel. All three devices use LCD screens but the iPad uses IPS (in plane switching) technology while both the Nexus 10 and the Tab 2 10.1 use the PLS (plane line switching) technology. Although they use similar principles, PLS is claimed to be brighter and have wider viewing angles than IPS.

**Table 1:** Resolution of screens (pixels), physical screen sizes (cm), pixels per cm and viewing distance (cm) required to permit testing 1.0 min of visual angle subtended per pixel (cm) for the 3 tablets tested.

	Resolution		Size		Pixels per	Viewing
	H	V	H	V	cm	Distance
iPad 3	2048	1536	19.6	15.1	103	33
Nexus 10	2560	1600	21.6	12.6	123	29
Tab 2 10.1	1280	800	22	13.8	58	59

**Table 2:** Warm up time (minutes) required for luminance to stabilize on screen after 3 different “off” periods.

	Off time		
	24hrs	10mins	1min
iPad 3	14	6	<1
Nexus 10	10	6	<1
Tab 2 10.1	8	4	<1

The devices were programmed by one of the authors (TMA), using an Apple Macbook Pro (©Apple Inc) running Adobe CS 5.5 with Flash and ActionScript 3.0 (©Adobe inc). Settings were adjusted so that the auto-adjust for brightness was switched off and mains power was connected during testing.

Luminance measurements were obtained by mounting the tablet in a vertical position 33 cm from a PR650 photospectroradiometer (Photo Research, Inc., Chatsworth, Ca., USA) with a spot size of 1°. The test image of a 400×400 pixel square stimulus was displayed in the center of the LCD. The set-up was housed in a psychophysics laboratory with all lighting switched off. The angle to the photometer was controlled using a precisely mounted protractor to determine the effects of viewing angle on luminance. Depending on the stimulus, contrast was specified in either Michelson or Weber contrast as follows.

Michelson contrast, for spatially repetitive stimuli such as gratings, was defined as (1):
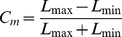
(1)where L_max_ represents the highest luminance and L_min_ represents the lowest luminance.

Weber contrast, for non-repetitive targets such as letters or spots, was defined as (2):
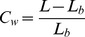
(2)where L represents luminance of the features and L_b_ represents the luminance of the background.

The following attributes were measured; gamma function, warm up time, screen uniformity, effects of reflectance

### Investigation 1; Gamma function assessment and calculation of range of programmable contrasts

Gamma (luminance intensity versus signal voltage) curves were assessed using the protocol described in [5]. Briefly, each tablet has an 8-bit grey scale programmable resolution. To calibrate it a central white square was presented at intervals of 8 programmable pixel intensities between 0 (black) and 255 (maximum white). Luminance was measured for each of these 33-programmed values. The values between each calibrated point were thereafter calculated by linear interpolation. These values were fed into a look-up table (LUT) and the curve produced by this LUT was fitted with a power function, the exponent of which was the gamma correction of the device. This relationship is defined by equation (3):
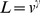
(3)where *L* represents the luminance of the display, *V* is the signal voltage and *γ* is the gamma.

The gamma curve from this investigation was used to derive the potential range of display of contrast targets on each device, taking into account the limits imposed by the relatively coarse luminance steps available in an 8-bit system.

The luminances that would be required to construct sinusoidal gratings or black on white letters of various mean luminances and contrasts were computed. Using the look-up table, these were rounded to the nearest available luminance and the practically achievable contrasts were derived. The available contrasts on each device were compared with those employed in two standard chart-based screening tests, the VisTech chart (Vision Sciences Research Corporation, San Ramon, California) and the Pelli Robson Contrast Sensitivity chart (Haag-Streit USA, Mason, Ohio).

Spatial independence was also tested. On an ideal screen the background luminance should not affect the target or foreground luminance. To assess any interaction between different areas of the screen 8 different target luminance levels where presented on 8 different background luminances to ensure contrast linearity for different backgrounds. The target was a centrally positioned square of 400 by 400 pixels.

### Investigation 2; Effective time to stability of display screen after switching on

The period of time from switching on the display to achieving stable luminance is an important characteristic. To assess this, the tablet was turned off overnight and when switched on the next day the central set luminance values were recorded over time until they stabilized. Device maximum luminance was assessed after 3 hours of on time and the criterion for stabilisation was taken as fluctuations of less than 0.5% around this max luminance. The device was then switched off for 1 minute. Luminance was measured after switching back on until it stabilized. This was then repeated for ‘off’ times of 1 and 10 minutes. Stability was also measured when the tablet was unplugged and run constantly on battery power until automatic power shut down.

### Investigation 3; Uniformity of luminance and contrast of targets at different angles of view

It is acknowledged that, in cathode ray tube (CRT) monitors, luminance varies towards the edges of the screen [6], typically by around 30% [7]. The tablet computers were programmed to display target squares (400 by 400 pixels in size) of 88% contrast and mean luminance as close to 200 cd/m^2^ as this contrast would allow.in each of the corners of the screen. Due to the variations in the LUT and gamma function between the devices and the limitation of the 8-bit system, to achieve contrast of 88% the achieved mean luminance was actually 210 cd/m for the iPad and Nexus 10 and 183 cd/m for the Tab 2 10.1. The photometer was angled towards each of them from a fixed central position 33 cm away from the screen. The objective was to simulate the possible clinical scenario of a patient positioned centrally to comfortably view both central and peripheral targets while also studying the potential effect of viewing targets at an angle to the screen. The effective contrasts for each of the conditions above were calculated from luminance values.

### Investigation 4; Effect of reflections and tablet screen angle from a light source on luminance and contrast of a target

A limitation of LCD screens is that they suffer from a fall-off of contrast with viewing angle. Due to the composition of different layers of filters and a reflective back surface on the screen, the three tablet screens exhibit strong reflections depending on the direction and intensity of the ambient illumination. These reflections can produce veiling luminance that will affect the contrast performance of the screen. While our measurements were taken in a light controlled environment, this may not be possible in clinics or if used as a portable testing device at home. To see how reflections from the screen impacted displayed contrast in an otherwise dark room, the tablets were placed in a light-controlled viewing booth. We measured the effect of adding a diffuse light source (mean luminance across tablet screen 60 cd/m^2^) in two positions, directly above the device (33 cms away) or positioned at a 45-degree angle to the screen (60 cms away). To further test the effect of viewing angle on contrast we tested the effect of angling the screen at 45-degrees to the normal viewing angle to determine the relationship between tilt angle and contrast. In a hand held device a tilt of 30–45 degrees is not an atypical viewing angle and it is important that its effect on displayed contrast is known.

## Results

### Investigation 1; Gamma function

**Figure 1:**
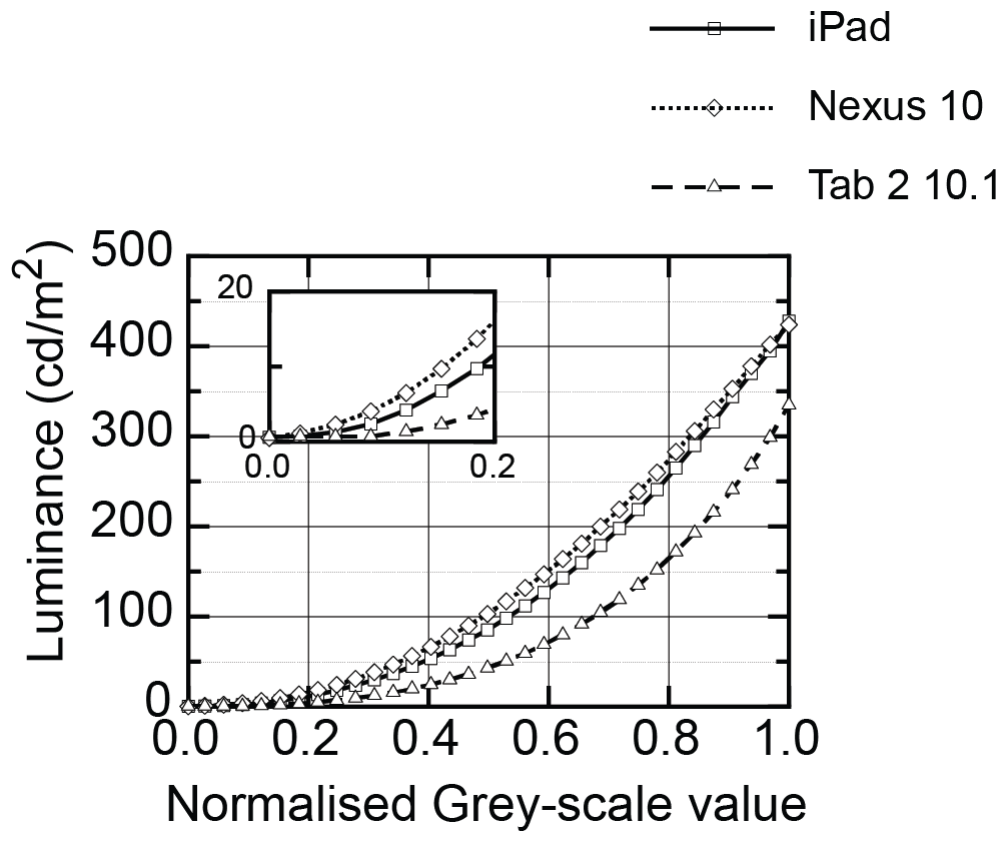
Gamma curves for the 32 measured luminances of a central area for the 3 tablets. Inset shows magnified view of the lowest 20% bit values. The programmable grey-scale values on the x-axis produce predictable and regular luminances according to the curve displayed.

Figure 1 shows the luminance curves for the three devices in cd/m^2^ for each normalized grey-scale bit value. The maximum brightness achieved with the iPad (428 cd/m^2^) and Nexus 10 (424 cd/m^2^) was very similar, while the Tab 2 10.1 was dimmer (335 cd/m^2^). The minimum brightness achieved for the iPad was 0.59 cd/m^2^, Nexus 10 0.49 cd/m^2^ and the Tab 2 10.1 0.71 cd/m^2^, resulting in respective contrast ratios of 725∶1, 865∶1 and 471∶1.

While LCD devices often exhibit an electro-optic response that is better modeled as a sigmoidal function [8], Bala and Sharma (2003) [9] found that many LCD manufacturers build correction tables into the video card that result in LCD responses mimicking the response of a CRT, with a power law whose exponent is about 2.0. To determine the gamma for the three devices the data in figure 1 was re-plotted on a log-log scale and a linear regression was fitted to the data. On a logarithmic scale a power function is linear, the slope indicating the exponent of the power function. Using this method the three devices tested here show a power-law relationship between the pixel intensities and output luminance. The gamma for each device was close to that of a typical CRT, which is between 2.35–2.55 [10], with values of 1.98 (iPad), 1.82 (Nexus 10) and 2.11 (Tab 2 10.1). These values also correlate well with the common LCD gamma of 2.0 found by [9].

**Figure 2:**
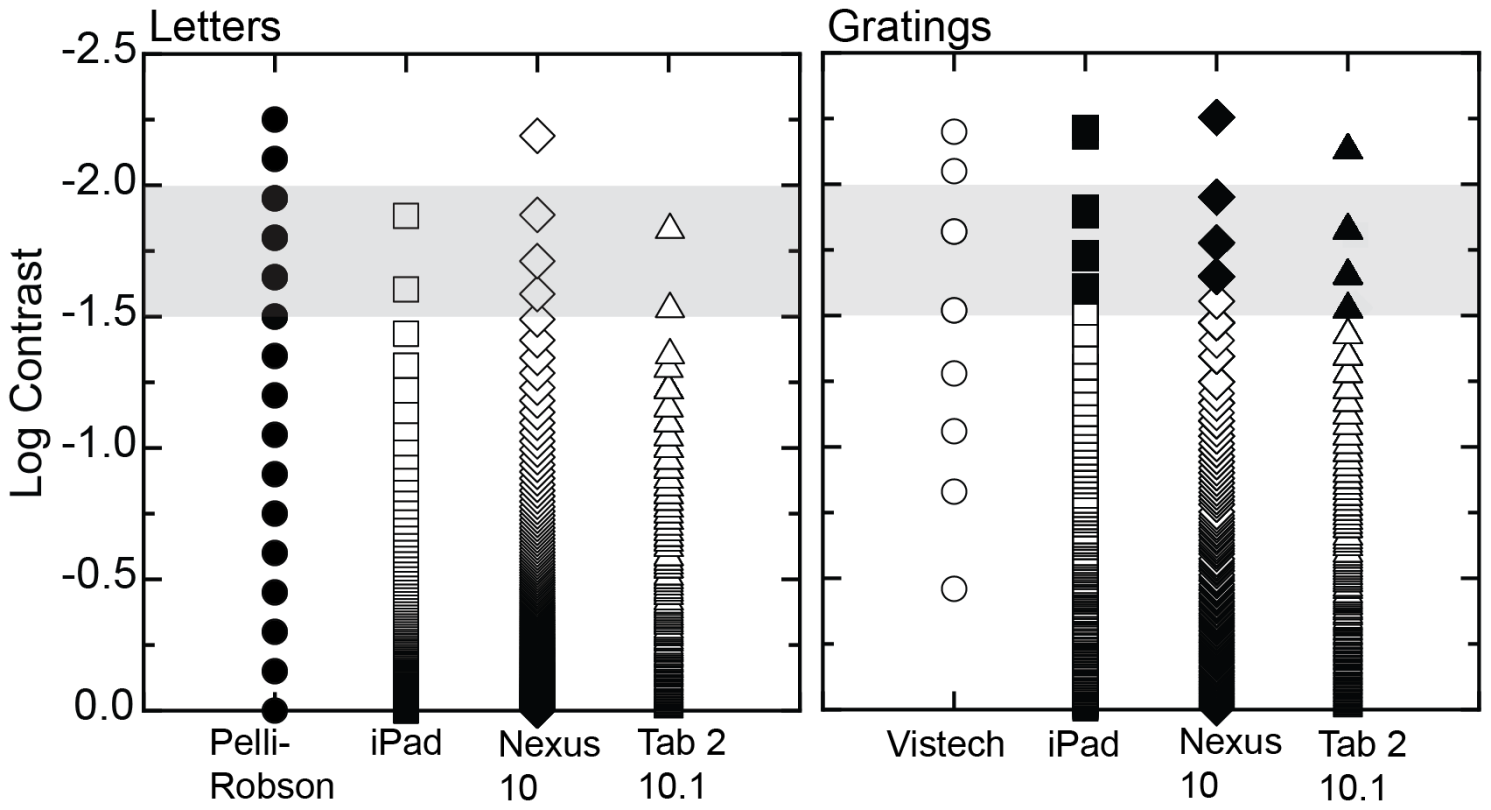
Available contrasts on the 3 tablet computers for black letters (Weber Contrast, left) and gratings (Michelson Contrast, right). Where contrast resolution meant that fewer than 5 luminance steps were available to draw the grating on the tablet computer, the symbols are shown in black. The grey band represents normal ranges for the Vistech and Pelli-Robson charts.

The derived look-up table was used to demonstrate an available contrast range for the devices to compare with the clinical standard Vistech and Pelli-Robson charts. Figure 2 illustrates the available contrast range for the devices and allows comparison with the standard chart-based vision tests. Weber contrasts are calculated for tablet screen letters (figure 2, left) and Michelson contrast for tablet screen gratings (figure 2, right). Background luminance for Weber contrast and mean luminance for Michelson contrast was taken as half of the maximum luminance of the device. This gave background luminance and mean luminance of 214, 212 and167.5 cd/m^2^ for the iPad, Nexus 10 and Tab 2 10.1 respectively. Where contrast resolution meant that fewer than 5 luminance steps were available to draw the grating on the tablet computer, the symbols are filled in black. All available Vistech contrasts are illustrated as published in the Vistech Manual and a report by [11]. The grey shaded region represents the normal ranges for the Vistech and Pelli-Robson charts.

As can be seen from figure 2, the Nexus 10 has the largest range of available contrasts and the Tab 2 10.1 the most limited. While none of the tablet screens are able to exactly match the very low levels of contrast of the Pelli-Robson chart (using Weber contrast for letters), they do have results that are close to these standards (figure 2, left), particularly the Nexus 10. The normal ranges (grey shaded areas in figure 2) are covered by the tablets and so it should still be possible to screen for contrast sensitivity deficits over a range of spatial frequencies. While resolution is limited in the normal range, the screens do display good resolution for higher contrasts, which can be particularly useful when measuring for defects.

**Figure 3:**
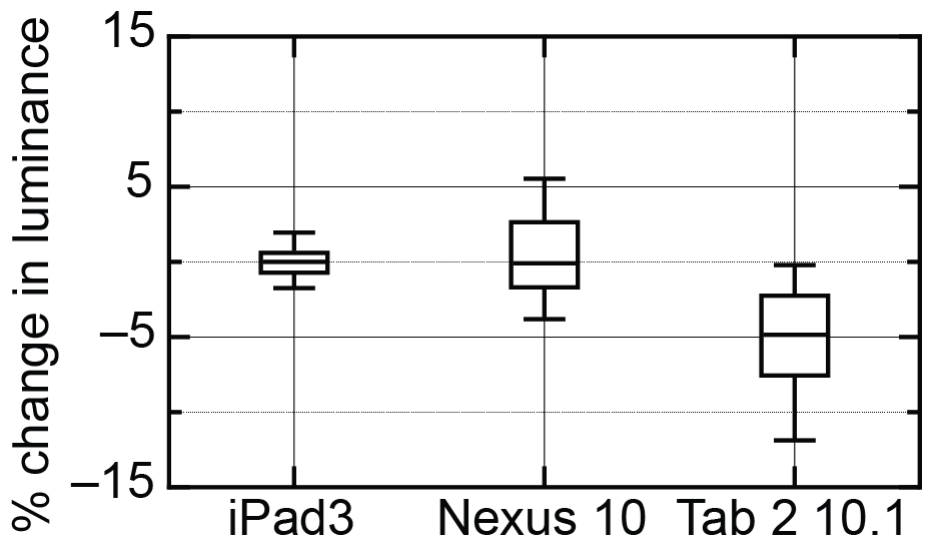
Box plots showing % change in luminance in the target when measured against 8 different backgrounds. Note that the mean change for the iPad and Nexus 10 are close to zero while mean change for the Tab 2 10.1 is −5%.

To check on the potential interaction between target and background, 8 different luminance levels were presented on 8 different background luminances. Ideally changing the background should have no impact on the target luminance, meaning contrast remains constant across backgrounds. Any differences in luminance are therefore errors. Figure 3 shows box plots displaying the percentage change in luminance in the target when the background was changed from a reference black background for each device. The iPad displays the least errors in luminance display. While for both the iPad and Nexus 10 the mean change in luminance was close to zero, the error was larger in the Nexus 10. The Tab 2 10.1 had the largest mean change in luminance (−5%) and the largest error of the three devices.

### Investigation 2; Stability of display screen after switch on

In our previous study [5] we found minimal changes in displayed contrast with varying screen warm up times in the iPad 3. Here we extended those investigations for the other 2 tablets tested. The data are shown below in table 2.

Even after being switched off for 24 hours, luminance only differed by 3 cd/m^2^ at most from the maximum achieved stable luminance and in all three tablets a stable luminance level was achieved within 15 minutes. Although there is an effect on luminance, contrast is a ratio and therefore is minimally affected. In comparison it is recommended that CRTs be allowed to warm up for 45 minutes before commencement of measurements [12]. Critically, the devices all recovered from a brief switch-off almost instantly. CRTs do not behave like this. If they are switched off for 1 minute, they the take almost as long to warm up as they do from cold. Luminance did not change when the devices were switched to battery power (power saving features were turned off) and also remained stable for all 3 devices as power was run down, right up until automatic shut down.

### Investigation 3; Uniformity of luminance and contrast of targets with different angles of view

**Figure 4:**
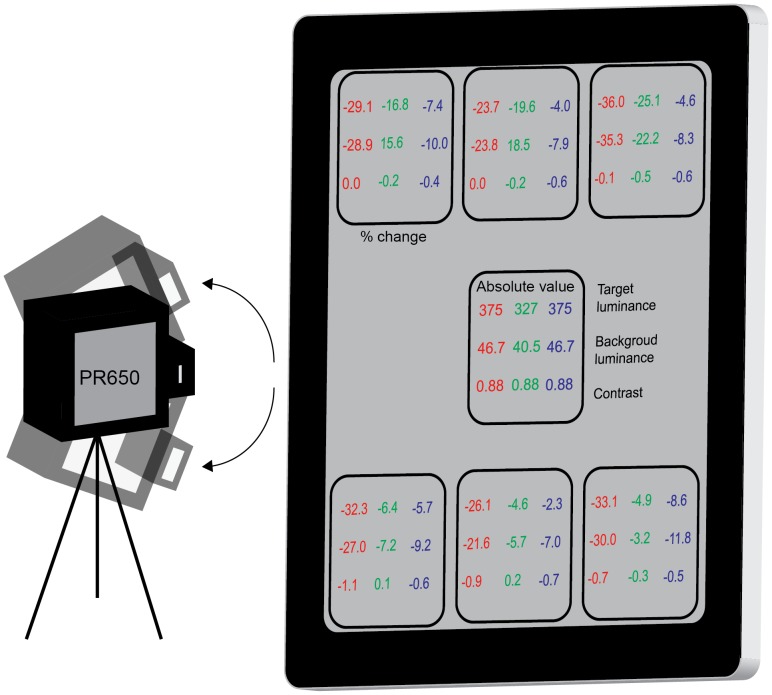
Errors in luminance (cd/m^2^) and contrast at different locations on the tablet screens measured if an observer were viewing all peripheral targets from a central location. Centre square shows target luminance (top value), background luminance (middle value) and contrast (bottom value) for the iPad (red values), Nexus 10 (green values) and Tab 2 10.1 (blue values). Peripheral squares display the percentage change in these values compared to the centre for each of the three tablets.

Figure 4 shows the errors in luminance and contrast respectively at different locations on the three tablet screens, with respect to the centre. Measurements were taken from a central position by rotating the photospectroradiometer, to replicate the effect of a subject focused on the central target and then glancing to the periphery. There was an expected reduction in luminance towards the four corners of the screen. It is clear that there is considerable non-uniformity across the screen, particularly in the iPad where the right hand regions of the screen show greater discrepancy than the left hand regions. As mentioned in the methods section, the Nexus 10 and Tab 2 10.1 are both based on PLS technology that is claimed to have better viewing angles than IPS screens such as the iPad screen. The data would agree with this, as luminance does not fall off as sharply in the periphery in both the Nexus 10 and the Tab 2 10.1. More pertinent to vision testing is the performance of the screens in displaying consistent contrasts. While the Nexus 10 had the smallest average contrast change over the peripheral areas of the screen (mean change 0.2% +/− 0.15), both the iPad (mean change 0.5% +/−0.47) and the Tab 2 10.1 (mean change 0.6% +/−0.1) have a minimal impact of contrast, at most around 1%. It is unlikely that this level of percentage change in calculated contrast would be clinically perceptible, as it is considerably less than one just-noticeable difference (JND).

### Investigation 4 – Effect of nearby light sources on luminance and contrast of a target

**Figure 5:**
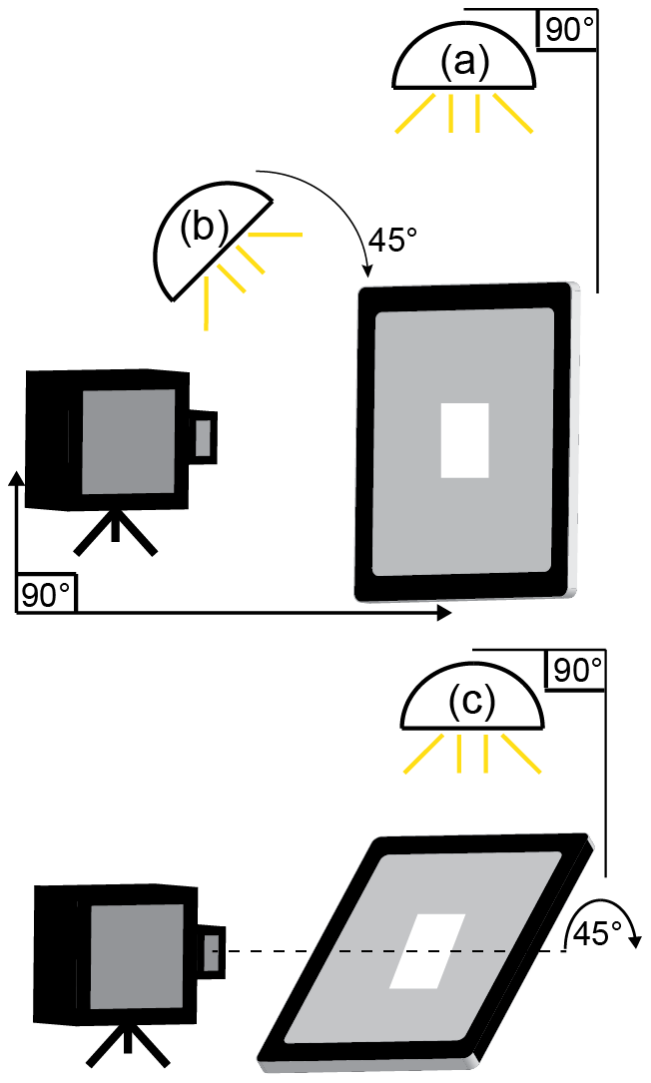
Setup for measurement of effect of reflections on displayed contrast. The light source was initially placed directly above tablet (condition a) and then moved to 45° from screen (condition b). The photospectroradiometer was placed directly in front of the display for measurement of luminance. In condition (c) the light source was placed directly above the tablet screen which was then rotated to 45° with respect to the photospectroradiometer.

Figure 5 illustrates the experimental setup for measurement of the effect of reflections on displayed contrast. Conditions (a) and (b) investigate the effect of light reflecting of a screen that is perpendicular to the line of gaze and condition (c) investigates the effect of rotating the screen with respect to the eye. Table 3 lists the measured luminance and contrast changes for all three tablets for these three conditions.

**Table 3:** Change in luminance (L) (%) and contrast (C) (%) under the three different conditions shown in figure 5 for the three devices.

	Condition A Screen 90° Light above		Condition B Screen 90° Light 45°		Condition C Screen 45° Light above	
	L	C	L	C	L	C
iPad 3	1.5	−0.4	0.3	−0.1	80.9	−44.9
Nexus 10	1.3	−0.3	0.3	0.2	15.4	−4.5
Tab 2 10.1	2.7	1.1	1.0	−0.1	84.9	−58.8

Major changes in contrast and luminance occurred only under condition C.

While the screen was perpendicular to the eye, the effect of adding a light source made clinically insignificant changes in luminance and contrast, even when the light source was placed at an oblique angle to the screen. Rotating the screen had a much larger effect in producing significant reductions, as it almost halved the displayed contrast in both the iPad and Tab 2 10.1 screens. The Nexus 10 screen performed better but still had a significant change in luminance when the screen was tilted and the impact of veiling luminance was to reduce contrast by 4.5%. All three screens are seriously hampered by rotation in the presence of a light source, producing changes in contrast that would be clinically significant during testing.

## Discussion

The current work reports on a series of measurements made on three different tablet screens to determine a procedure for optimizing their performance for vision testing and some physical characteristics of the screens and their impact on potential vision tests. The key findings are discussed below.

### Spatial resolution

It would be desirable for a device to be able to test at least 1 min of arc (6/6 or 20/20 Snellen acuity) at a reasonable testing distance. The most common reading distance is typically 33–40 cms; at this distance, both the Nexus 10 and iPad would allow for 6/6 or even 6/5 vision testing (Nexus 10 at 33 cms and iPad if held at 40 cms). However, the poorer screen resolution of the Tab 2 10.1 only allows it to achieve 1 min of arc resolution if held at 60 cms away. This poses two disadvantages. One is that most near corrections are set for a reading distance of 33–40 cms and so, in older presbyopic patients, the Tab 2 10.1 is at an intermediate distance that may appear blurred with either their near or distance correction. The second disadvantage is that, in a touch screen display, it may be too far away for someone to easily and accurately reach out and touch for input. We therefore conclude that a tablet with a screen resolution that has at least 100 pixels per cm would be desirable for vision testing.

### Gamma correction, contrast and effect of background

As would be expected, all three devices had differences in their luminance range and the determined LUT. When individually calibrated, the Nexus 10 had the largest range of contrasts for testing while Tab 2 10.1 had the smallest. However the contrast ranges for all three were quite similar and, while not matching the traditional charts for very low levels of contrast, would allow for testing in the normal range for contrast sensitivity. Indeed, for all three tablets calibration of the gamma function curves is essential to allow for precise and predictable contrast testing. Note that, for a screening test, this does not require recourse to such contrast-improvement techniques as dithering or bit-stealing [13],[14], though these methods could be implemented to further increase the contrast range if desired. Such techniques were employed successfully by [3] using an iPad to measure low contrast thresholds. In fact, such approaches are vital if, rather than being used for screening, an 8-bit device is to be used to measure contrast thresholds. The protocol is easily implemented and our recommendation to programmers would be to incorporate it even in a screening tool.

Spatial independence is an important characteristic of a screen for vision testing, as it is important that the background does not influence the target luminance to ensure correct contrast reproduction. The iPad3 had the best performance of the three devices. The Nexus 10 had only small errors but the Tab 2 10.1 was least consistent with a mean error of −5%. Such a difference would be clinically significant and so the Tab 2 10.1 does not demonstrate adequate spatial independence to allow for clinical vision testing.

### Warm up characteristics

CRT screens typically require 45 minutes of warm up time before luminances stablise [12]. Our tests showed that all three devices could be used with confidence within a few minutes of turn on from cold. For total luminance stability a 15-minute warm up period is required for all devices, much quicker than CRT screens.

### Screen luminance uniformity

The LUT and corresponding gamma curve for each screen was produced based on a central target. To determine whether this central calibration would be adequate for peripheral targets uniformity across the screen was determined. While all 3 tablets showed changes in luminance across different areas of the screen when viewed from the central position, contrast remained stable for all 3 devices. The errors were all under 1.1% contrast and so would not be clinically significant.

### Effects of surround illumination and viewing angle

For a potential hand held and possibly home testing device such as a tablet screen, the effect of reflections and tilting of the screen on displayed luminance and contrast is important. Our testing showed that the combination of reflections from light sources and tilting of the tablet screen with reference to the viewing plane can have clinically significant effects on observed luminance and contrast (particularly with screen tilt) for all tested tablet screens. This is in agreement with [4], who found that visual acuity measures were unreliable until the iPad screen was mounted perpendicular to the floor and an antiglare screen attached. While [4] did not specify the antiglare screen used or its characteristics in terms of reduction of glare, our measurements indicate that they key property for reflections is the angle of the screen to the viewer, with screen tilt very debilitating for contrast stability

## Conclusions

The recent publications using an iPad for visual testing [3], [4] demonstrate that such devices are ready for adoption in the clinical space. The main point to emerge from the investigations conducted both here and in [5] is that measuring the gamma function to produce an LUT specific to the device is crucial. It determines the luminance and contrast range possible for the device and then allows for complete control and great precision over displayed contrast and luminance. Calibration of each screen therefore is important for the integrity of the measurements obtained with the device. Here we have outlined a protocol for measuring the gamma function and LUT that can be applied by others. Individual device calibration will ensure that any variances between devices, even of the same manufacturer and model, are taken into account and enable accurate presentation of test stimuli. The second point to emerge is that reflectance from external light sources will radically affect the measurements. While [4] used a darkened room, it is undesirable to recommend the computers be used for vision testing without some form of surround lighting for safety reasons and so it is important that the device be mounted in some form of surround to control both viewing angle artifacts and control of ambient illumination. Finally, it is important to recognize that, although luminance is not uniform across the screens, this variability has little impact on specified contrast, because background/target luminance ratios are remarkably linear for the devices tested.
